# Psychological Support and Return-to-Sport Readiness After Injury: Evidence from Student-Athletes

**DOI:** 10.1192/j.eurpsy.2026.11669

**Published:** 2026-07-31

**Authors:** M. Mouldi, S. Ben Abed, A. Othman, L. S. Chaibi, H. Ghabi, H. Nefzi, F. Ellouze

**Affiliations:** 1 Department of Psychiatry G, Razi Hospital, Manouba; 2 Faculty of Medicine, University of Monastir, Monastir, Tunisia

## Abstract

**Introduction:**

Returning to sport after injury is not solely a physical process. Psychological readiness and fear of re-injury are key determinants of a safe and confident return. Yet, mental health support during rehabilitation remains inconsistently integrated into recovery programs.

**Objectives:**

This study aimed to determine whether student-athletes who received mental health support during rehabilitation exhibited higher psychological and physical readiness to return to sport compared to those who did not. Secondary objectives included exploring associations between mental health support, kinesiophobia, and physical readiness.

**Methods:**

A cross-sectional study was conducted among 50 student-athletes from the Higher Institute of Sport and Physical Education (ISSEP) who had returned to full sport participation within the past year following a musculoskeletal injury that required at least four weeks of rehabilitation. Participants completed questionnaires assessing demographics, injury history, and perceived mental health support, along with validated scales: the Anterior Cruciate Ligament–Return to Sport after Injury (ACL-RSI) for psychological readiness, the Tampa Scale for Kinesiophobia (TSK-11) for fear of re-injury, and the Oslo Sports Trauma Research Center (OSTRC- O2) questionnaire for physical readiness. Data were analyzed using SPSS Statistics version 26.

**Results:**

Among 50 student-athletes (mean age ≈ 22 years; 58% female), 64% reported receiving adequate mental or emotional support during rehabilitation. Athletes who received support showed significantly higher psychological readiness to return to sport (ACL-RSI = 39.0 ± 24.5) than those without support (21.9 ± 15.3; *t*(37.8) = 3.04, *p* = 0.004, *d* = 0.79). No significant group differences were observed for kinesiophobia (TSK = 40.0 ± 8.7 vs. 42.4 ± 8.6; *p* = 0.35) or physical readiness (OSTRC-O2 = 10.0 ± 4.5 vs. 11.3 ± 5.7; *p* = 0.40). Psychological readiness was moderately correlated with perceived support (*r* = 0.36, *p* = 0.01), while TSK and OSTRC were positively associated (*r* = 0.43, *p* = 0.002), suggesting that higher fear of re-injury relates to greater perceived physical limitation.

**Conclusions:**

Student-athletes who received mental or emotional support during rehabilitation showed markedly greater psychological readiness to return to sport, independent of physical or kinesiophobic factors. These findings highlight the importance of integrating structured mental health support into rehabilitation to optimize return-to-sport outcomes. However, the cross-sectional design, small sample, and self-reported data limit causal inference. Future longitudinal studies should clarify causal pathways and identify which support components provide the greatest benefit.

**Disclosure of Interest:**

None Declared

